# P01-031 – Anakinra for colchicine resistant FMF

**DOI:** 10.1186/1546-0096-11-S1-A35

**Published:** 2013-11-08

**Authors:** I Ben-Zvi, OL Kukuy, M Lidar, O Feld, O Perski, S Kivity, P Langevitz, B Pistrom, A Livneh

**Affiliations:** 1 Heller Institute of Medical Research, Sheba Medical Center, Ramat-Gan, Israel; 2 Swedish Orphan Biovitrum, (SOBI), Stockholm, Sweden

## Introduction

About 10-20% of Familial Mediterranean Fever (FMF) patients do not achieve complete remission, due to colchicine resistance or sensitivity. Disease control in these patients is still an unmet challenge. Case reports and a small case control study, suggest a role for interleukin 1 beta blockage, particularly anakinra, in the management of this limitation of colchicines.

## Objectives

To embark on a study, evaluating the efficacy and safety of anakinra in the treatment of colchicine refractory FMF.

## Methods

We plan to include patients, agreeing with clinical and genetic diagnosis of FMF, who suffer from FMF attacks, at least once per month, in one of the sites commonly involved by FMF (Chest, abdomen, lower extremity large joints, and skin), despite treatment with colchicine 2 mg/day or less (in case of colchicine intolerance). Involvement with other diseases relevant (vasculitis, spondyloarthropathy, Behcet's disease, etc.), or irrelevant (rheumatoid arthritis, SLE, etc.), to FMF, or possible non compliance, will serve as exclusion criteria. The study is planned to continue for 4 months per patient, in which patients will receive anakinra (s.c. 100 mg/day, 25 patients), or control drug (anakinra vehicle, same volume, same package, 25 patients). Randomization will be sequential for a predetermined order of the interventional drug (anakinra or vehicle), for which the study team will be blinded. Analysis of the results will be performed by an external company. Anakinra effect will be compared to placebo effect by computing the reduction of number of attacks per each patient. Secondary outcome include reduction of severity of attacks.

## Results

No results are yet available. The study is an investigator initiative project, with sponsorship of the manufacturing drug company. Measurements were taken to avoid any bias in the performance of the study or interpretation of the results. It is expected that the whole study will continue 2 years.

## Conclusion

A favorable anakinra effect in the prevention of FMF attacks in colchicine failure, supported by ample case reports is expected to be confirmed by the present controlled double blinded study by mid 2015.

## Disclosure of interest

None declared.

